# Impact of Small-Quantity Lipid-Based Nutrient Supplements on Pubertal Status of 9–13-Year Olds: A Follow-Up Study of the iLiNS-DYAD-Ghana Trial

**DOI:** 10.1016/j.cdnut.2024.104458

**Published:** 2024-09-26

**Authors:** Helena Nti, Seth Adu-Afarwuah, Brietta M Oaks, Elizabeth L Prado, Charles D Arnold, Paul D. Hastings, Amanda E Guyer, Kathryn G Dewey, Benjamin Amponsah, Helena J Bentil, Mavis Osipi Mensah, Ebenezer Adjetey, Xiuping Tan, Lois Maame Donkor Aryee, Fatimah Bintu Ayete Labi, Adom Manu

**Affiliations:** 1 Department of Nutrition and Food Science, University of Ghana, Accra, Ghana; 2 Department of Sports and Exercise Medical Sciences, University of Health and Allied Sciences, Ho, Ghana; 3 Department of Nutrition, University of Rhode Island, Kingston, RI, United States; 4 Institute for Global Nutrition, Department of Nutrition, University of California, Davis, Davis, CA, United States; 5 Center for Mind and Brain, University of California Davis, Davis, CA, United States; 6 Department of Psychology, University of California Davis, Davis, CA, United States; 7 Department of Human Ecology, University of California Davis, Davis, CA, United States; 8 Department of Psychology, University of Ghana, Accra, Ghana; 9 Department of Population, Family and Reproductive Health, School of Public Health, University of Ghana, Accra, Ghana

**Keywords:** first 1000 d, SQ-LNS, birth weight, pubertal development, adolescents, Ghana

## Abstract

**Background:**

Early and delayed puberty are both associated with adverse health and psychosocial outcomes.

**Objectives:**

We assessed the impact of provision of small-quantity lipid-based nutrient supplement (SQ-LNS) to mothers during pregnancy and 6 mo postpartum and to their children aged 6–18 mo, on pubertal status.

**Methods:**

This study was a follow-up to a partially double-blind randomized controlled trial. At ≤20 wk, 1320 females were randomly assigned to receive daily: iron and folic acid during pregnancy and placebo 0–6 mo postpartum; or multiple micronutrients during pregnancy and 0–6 mo postpartum; or SQ-LNS during pregnancy and 0–6 mo postpartum and to their children from 6 to 18 mo. We re-enrolled 966 and 919 children at 9–11 y and 11–13 y, respectively. We calculated a total pubertal status score based on the Petersen Pubertal Development Scale (PDS) to assess growth spurt, skin changes, body hair, facial hair, voice break, breast development, and menstruation. Pubertal status was regressed on child’s age to generate age-adjusted PDS z-scores (aPDSZ); we performed interaction and mediation analyses.

**Results:**

Mean ± standard deviation aPDSZ did not differ between the SQ-LNS and non-LNS groups at 9–11 y (0.01 ± 0.95 compared with –0.01 ± 0.98; *P* = 0.958) but was more advanced in the SQ-LNS group at 11–13 y (0.07 ± 1.04 compared with –0.04 ± 0.98; *P* = 0.049) in the adjusted model. The effect of SQ-LNS varied by sex (*P*-interaction = 0.003) and household asset index z-score (*P*-interaction = 0.002): Puberty was more advanced in the SQ-LNS compared with non-LNS group among females (*P* = 0.007) but not males (*P* = 0.877), and within lower (*P* = 0.002) than average (*P* = 0.436) and higher (*P* = 0.332) socioeconomic households.

**Conclusion:**

Provision of SQ-LNS during the first 1000 d of life advanced pubertal status among females.

**Trial registration number:**

This trial was registered at clinicaltrials.gov as NCT00970866 (https://clinicaltrials.gov/ct2/show/record/NCT00970866).

## Introduction

Pubertal development is controlled by the hypothalamic pituitary gonadal (HPG) axis [1]. The HPG axis initiates development of the reproductive system at mid-gestation, until after the first year when it becomes inactive [2]. The HPG axis is then reactivated when pubertal maturation begins [3]. This timetable of changes suggests possible prenatal intervention in the reawakening of the HPG axis during puberty. The pubertal development process may be affected by environmental stimuli and cellular metabolites including those of sex hormones [4].

In animals, growth and age of pubertal onset can be manipulated in ways that include changing diets and injecting hormones during the prenatal period. Evidence from such studies suggests that fetal nutrient and/or hormonal exposures may affect fetal development. For example, there is an increased risk of fetal growth restriction (FGR) in suboptimal environments [5], such as those with poor maternal nutrition. This may lead to low birth weight and small-for-gestational age (SGA). If there is catch-up growth (reflecting increased growth rate after a period of growth delay [6] during early childhood after such exposures, the growth and its related metabolic alterations may expedite the reactivation of the HPG axis and pubertal onset [7].

Several nutrients such as fatty acids, iron, iodine, zinc, and B-vitamins play important roles in nerve tissue functions and metabolic processes [8,9], which may affect the activity of the HPG axis and hence, pubertal development. The brain is lipid rich with its structural components dominated by long-chain PUFA such as DHA [10]. Therefore, insufficient intake of the essential fatty acids alpha-linolenic acid and linoleic acid, which can be converted into DHA and arachidonic acid, respectively, may contribute to impaired brain function [10,11]. Prenatal malnutrition may alter the expected timeline and function of the HPG axis, and consequently shift pubertal development.

In low-income settings, cereals and tubers are common staples consumed by pregnant and lactating females; these staples are of low nutrient quality [12]. Multiple micronutrient (MMN) deficiencies, associated with consumption of diets dominated by these foods, are associated with poor maternal and child outcomes such as FGR and poor growth and development among children [[13], [14], [15]]. The International Lipid-based Nutrient Supplements (iLiNS) Project team developed small-quantity lipid-based nutrient supplements (SQ-LNS) (20 g/d) for enriching the usual diets of pregnant and lactating females and infants with micronutrients, essential fatty acids, and small amounts of energy (118 kcal/d) and high-quality protein (2.6 g/d) during the first 1000 d. At least 16 trials have been conducted in 10 countries in which versions of SQ-LNS were provided either to infants only during the period 6–23 mo of age [[16], [17], [18], [19], [20], [21], [22], [23], [24], [25], [26], [27]], or to both mothers and infants [[28], [29], [30], [31]] during pregnancy, lactation, and infancy. In meta-analyses [[32], [33], [34]], the provision of SQ-LNS to infants aged 6–24 mo was associated with positive anthropometric outcomes, including a reduction in the prevalence of stunting/severe stunting, wasting/severe wasting, and underweight. There was a lower prevalence of anemia, when compared with the provision of no intervention.

Early pubertal timing has been associated with development of metabolic syndrome and cancers of the reproductive system [35] in later life, whereas late pubertal timing has been linked to psychosocial difficulties [36]. In Sub-Saharan Africa, previous studies have shown associations of stunting and underweight with later pubertal development [[37], [38], [39]]. On the other hand, there is the increasing prevalence of earlier onset of puberty over the past 2–3 decades, parallel to the global increasing prevalence of obesity. In Ghana, prior studies have shown a decline in age at menarche by 9–15 mo with obesity and high socioeconomic status as predictive factors among urban schoolgirls [38,40]. These suggest that nutritional factors and their corresponding endogenous metabolic and hormonal signals play relevant roles in pubertal development [41], which is consistent with studies showing positive associations of anthropometric measurements with pubertal development [42,43].

In the iLiNS-DYAD trial in Ghana, we demonstrated that SQ-LNS provided during the first 1000 d positively impacted birth size as well as subsequent growth [44,45] and socioemotional [45,46] outcomes. In this study, we assessed whether there were intervention group differences in pubertal status at 9–11 and 11–13 y. We hypothesized that the SQ-LNS group would be more advanced in pubertal development than the non-LNS group at these ages [35]. We also tested whether sex, household asset index, or birth order moderated the effect of the intervention on pubertal stage. We did not set a priori hypotheses for effect modification. Finally, we investigated whether the effect of the intervention on pubertal stage was mediated by birth weight for gestational age z-score or BMI z-score (BMIZ) at 4–6, 9–11, and 11–13 y.

## Methods

### Summary of the original iLiNS-DYAD trial

The iLiNS-DYAD Ghana study (2009–2014) was a randomized, partially double-blind, controlled trial that compared 3 nutrient supplements. The trial was conducted in peri-urban settlements in the Yilo Krobo and Lower Manya Krobo districts of the Eastern region of Ghana, ∼70 km north of Accra (the capital of Ghana). Primary caregiver written informed consent was obtained prior to data collection. Each caregiver was visited at home and visited our project office, whereas we collected data on sociodemographic information using interviewer-administered questionnaires. Details of the trial have been previously reported elsewhere [44,47] and are summarized herein.

### Study participants and procedure

In the main iLiNS-DYAD randomized controlled trial (RCT), females were eligible to participate if they were ≥18 y of age and ≤20 wk gestation. Females were excluded for the following reasons: antenatal card indicated HIV infection, asthma, epilepsy, tuberculosis, or any malignancy; known milk or peanut allergy; not residing in the study area; intention to relocate within the next 2 y; unwillingness to consent to participate, receive home visits from fieldworkers or take the study supplement; and participation in another trial.

Pregnant females attending antenatal clinics in 4 main health facilities in the study area were recruited from December 2009 to December 2011. Eligible females were visited at home and those who met the inclusion criteria and provided informed consent were scheduled for a clinic visit for baseline assessments. Enrolled females were randomly assigned to 1 of 3 supplementation groups (Table 1) [48]: *1*) daily 60 mg iron and 400 μg folic acid (IFA) during pregnancy, and 200 mg calcium (Ca) only during the first 6 mo postpartum, with no supplementation for offspring during infancy; *2*) daily MMN (1–2 Recommended Dietary Allowance of 18 vitamins and minerals) during pregnancy and the first 6 mo postpartum, with no supplementation for offspring during infancy, and *3*) daily 20 g SQ-LNS during pregnancy and the first 6 mo postpartum (SQ-LNS during pregnancy and lactation contained similar vitamin and mineral content as the daily MMN, plus calcium, phosphorous, potassium, magnesium, and essential fatty acids), with SQ-LNS for offspring (20 g with 22 vitamins and minerals with concentrations based on Recommended Nutrient Intakes for infants) from 6 to 18 mo of age. Participants were aware whether they received SQ-LNS or a capsule but were blind to whether they received IFA or MMN. Field workers who assessed outcomes and collected other data were blind to the intervention groups.

**TABLE 1:** Nutrient and energy contents of supplements used in the iLiNS trial in Ghana.

Ration per day	IFA	MMN	Maternal SQ-LNS	Child SQ-LNS
	1 capsule	1 capsule	20-g sachet	20-g sachet
Total energy (kcal)	0	0	118	118
Protein (g)	0	0	2.6	2.6
Fat (g)	0	0	10	9.6
Linoleic acid (g)	0	0	4.59	4.46
α-Linolenic acid, (g)	0	0	0.59	0.58
Vitamin A (μg RE)	0	800	800	400
Vitamin C (mg)	0	100	100	30
Vitamin B-1 (mg)	0	2.8	2.8	0.3
Vitamin B-2 (mg)	0	2.8	2.8	0.4
Niacin (mg)	0	36	36	4
Folic acid (μg)	400	400	400	80
Pantothenic acid (mg)	0	7	7	1.8
Vitamin B-6 (mg)	0	3.8	3.8	0.3
Vitamin B-12 (μg)	0	5.2	5.2	0.5
Vitamin D (mg)	0	10	10	5
Vitamin E (mg)	0	20	20	6
Vitamin K (μg)	0	45	45	30
Iron (mg)	60	20	20	6
Zinc (mg)	0	30	30	8
Copper (mg)	0	4	4	0.34
Calcium (mg)	0	0	280	280
Phosphorus (mg)	0	0	190	190
Potassium (mg)	0	0	200	200
Magnesium (mg)	0	0	65	40
Selenium (μg)	0	130	130	20
Iodine (μg)	0	250	250	90
Manganese (mg)	0	2.6	2.6	1.2

Abbreviations: IFA, iron and folic acid capsule; iLiNS, international lipid-based nutrient supplement; MMN, multiple micronutrient supplement capsule; SQ-LNS, small-quantity lipid-based nutrient supplement.

Information from table previously published [48].

### Follow-up study design and methods

Using participants’ addresses and telephone contacts from the original iLiNS-DYAD-Ghana trial, caregivers with surviving children were traced and invited to participate in this study. We obtained ethical approval for the current follow-up study from the Institutional Review Board of the University of California Davis (IRB ID: 1489918) and the Ghana Health Service Ethical Review Committee (GHS-ERC: 027105119). Child written informed assent was obtained prior to data collection. Each child visited our project office while we collected data on pubertal stage using interviewer-administered questionnaires. At the 4–6-y follow-up, weight and height measurements were taken by trained anthropometrists as reported previously [49], and these measurements were repeated at 9–11 and 11–13 y. Pubertal status was assessed at 9–11 y (2020–2021) and 11–13 y (2022–2023) of age. Our targeted enrollment of 966 provided 80% power to detect a small effect size, that is, a mean difference between 2 groups of >0.2 SD for continuous outcomes at a significance level of *P* < 0.05.

### Assessment of outcome

Participants responded to each item on the Petersen Pubertal Development Scale (PDS) score [50] using a 4-point Likert scale that described the stage of 5 pubertal milestones each, for boys and girls. On the scale, 1 was defined as pubertal development not yet begun, 2 as barely started, 3 as definitely underway, and 4 as completed. In girls, we determined PDS score by self-report of breast development, menarche, growth spurt, skin changes, and body hair. In boys, we determined pubertal development by self-report of voice break, facial hair, growth spurt, skin changes, and body hair. A total score ranged from 5 to 20. We conducted a pilot study of 30 caregiver-child dyads in our study sample to assess test-retest reliability of the PDS score. Our pilot study showed that the 1-wk test-retest reliability (*N* = 30; child report: *r* = 0.72; caregiver report: *r* = 0.70) of the PDS was good [50,51]. We selected the child report, which had a higher test-retest reliability score, for assessing pubertal status. In addition, we adjusted the PDS score by age in a regression model, computed residuals and standardized to calculate age-adjusted PDS z-score (aPDSZ) as our outcome.

### Statistical analysis

We posted a statistical analysis plan to Open Science Framework (https://osf.io/7j368) before conducting analyses. All analyses were performed using R version 4.3.1 (2023–06–16 ucrt, R Foundation for Statistical Computing). All tests were 2-sided, at 95% confidence interval (CI) and a 5% level of significance following a complete case intention-to-treat framework. We planned to compare SQ-LNS compared with non-LNS (IFA + MMN) groups. Nevertheless, we first performed a sensitivity analysis comparing the IFA and MMN groups to confirm that combining the groups was reasonable. We examined whether children in the SQ-LNS and non-LNS groups were similar in baseline characteristics using descriptive statistics for each intervention group. To evaluate potential bias in the sample, we compared baseline characteristics between the sample included in the analyses and the sample enrolled in the main trial but lost to follow-up, using t tests for continuous variables and χ^2^ tests for categorical variables.

To test our hypothesis that the SQ-LNS group was more advanced in pubertal stage than the non-LNS group at 9–11 and 11–13 y of age, we examined the difference between the SQ-LNS and non-LNS groups using analysis of covariance (ANCOVA) in minimally adjusted (with child sex) and covariate adjusted models. The prespecified covariates listed above were tested for associations with the aPDSZ. Significant (*P* < 0.10) covariates were included in the adjusted ANCOVA model. Prespecified covariates were identified from previous studies [48,52] and included sociodemographic background characteristics at baseline: maternal age, years of maternal education, maternal marital status, household asset index, household food insecurity, household improved water, household toilet facility, maternal prepregnancy BMI (kg/m^2^), maternal height, maternal hemoglobin concentration, season of birth, birth order, and parity.

In exploratory analyses, we tested potential effect modification by 3 prespecified variables: baseline household asset index, child sex, and birth order. We tested the interaction between each potential effect modifier and intervention groups in ANCOVA models. Significant interactions (*P* < 0.10) were further examined with stratified analyses and regions of significance in stratified analyses [53].

We tested potential mediation of exposure effects by: *1*) birth weight measured in grams and used to calculate birth weight for gestational age z-score, based on INTERGROWTH norms, to account for variations in birth weight by gestational age and child sex; *2*) childhood nutritional status indexed by BMIZ at ages 4–6, 9–11, and 11–13 y, standardized using WHO norms. Potential mediators that were associated with SQ-LNS or aPDSZ at *P* < 0.10 were included in causal mediation analyses using the mediation package in R [54].

## Results

**FIGURE 1:**
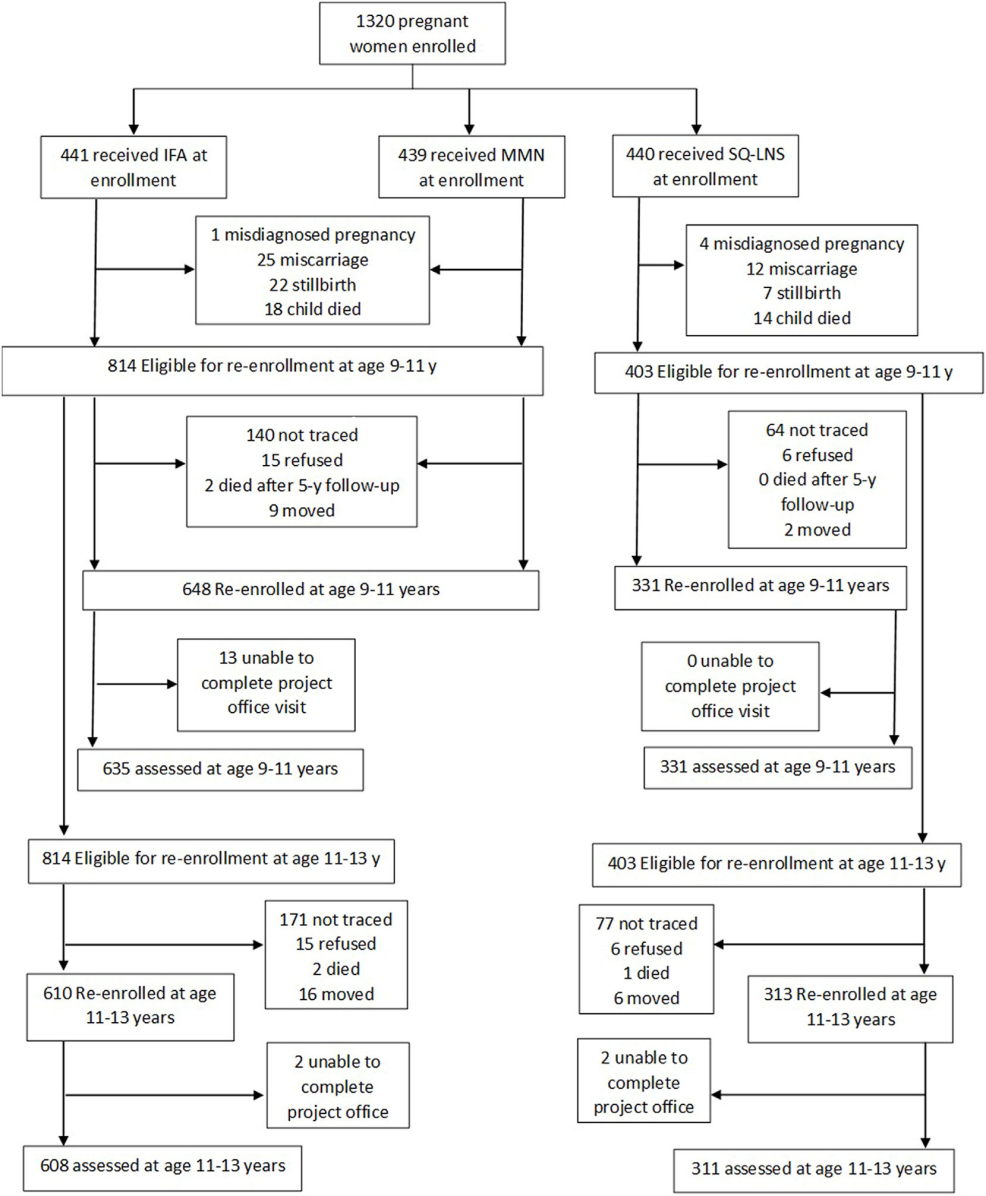
Flowchart of child’s eligibility, enrollment and data collection.

Out of 1320 mothers enrolled during the main trial, 1228 (93%) children were born live. A total of 1217 children were eligible to participate in the follow-up study at 9–11 y; 966 children participated in the pubertal development assessment including 635 in the non-LNS group and 331 in the SQ-LNS group. At 11–13 y, 919 children participated in the pubertal development assessment including 608 in the non-LNS group and 311 in the SQ-LNS group (Figure 1). The 966 and 919 included in the analysis were similar to the 354 and 401 enrolled in the main trial, respectively, but lost to follow-up regarding most background characteristics, such as baseline maternal education, food security, and BMI (Supplemental Table 1).

### SQ-LNS and non-LNS group comparisons

Sensitivity analysis showed no difference between IFA and MMN groups (*P* > 0.10) at both 9–11-y and 11–13-y follow-up; hence, we proceeded to analyze by a 2-group comparison, SQ-LNS and non-LNS groups (IFA+MMN). Table 2 [55] presents selected maternal and child characteristics. The 2 groups generally did not differ significantly in the 11 maternal baseline characteristics presented except for baseline household asset index z-score. At 9–11 y (*P* = 0.019) and 11–13 y (*P* = 0.019), the SQ-LNS group had lower asset index z-score than the non-LNS group.

**TABLE 2:** Selected maternal and child characteristics at 9–11-y follow-up.

Variable	SQ-LNS (*n* = 331) Mean (SD) or % (*n*/total)	Non-LNS (*n* = 635) Mean (SD) or % (*n*/total)
Maternal characteristics at baseline		
Age (y)	27.0 (5.5)	26.8 (5.4)
Education (y)	7.6 (3.8)	7.7 (3.6)
Married or cohabiting, % (*n*/*N*)	91.8 (304/331)	94.0 (596/634)
Household asset index z-score^1^	–0.07 (0.97)	0.09 (0.97)
Household food secure, % (*n*/*N*)	60.3 (199/330)	56.98 (359/630)
Household improved water source, % (*n*/*N*)	99.1 (328/331)	98.1 (621/633)
Household toilet facility, % (*n*/*N*)	97.6 (322/330)	97.6 (618/633)
Height (cm)	159.1 (5.4)	158.8 (5.9)
Prepregnancy BMI^2^ (kg/m^2^)	24.9 (4.5)	24.3 (4.4)
Hemoglobin concentration (g/L)	111.5 (11.2)	111.4 (12.6)
Primiparous, % (*n*/*N*)	33.7 (102/303)	31.7 (201/635)
Child characteristics		
Dry season^3^, % (*n*/*N*)	50.5 (167/331)	48.9 (310/634)
First born, % (*n*/*N*)	30.8 (102/331)	31.7 (201/634)
Females, % (*n*/*N*)	51.7 (171/331)	51.7 (328/634)
Child age at 4–6-y follow-up (y)	4.9 (0.6)	4.9 (0.5)
Child age at 9–11-y follow-up (y)	9.9 (0.5)	9.9 (0.5)
Birth weight for gestational age z-score	–0.41 (0.97)	–0.61 (0.95)
Child BMIZ at 4–6 (y)	–0.55 (0.81)	–0.58 (0.81)
Child BMIZ at 9–11 (y)	–0.43 (1.18)	–0.50 (1.16)
PDS score	7.5 (1.1)	7.5 (1.1)

Abbreviations: non-LNS, iron and folic acid + multiple micronutrient groups; PDS, Petersen Pubertal Development Scale; SQ-LNS, small-quantity lipid-based nutrient supplement.

1 Proxy indicator for household socioeconomic status constructed for each household based on ownership of a set of assets (radio, television, etc.), lighting source, drinking water supply, sanitation facilities, and flooring materials. Household ownership of this set of assets is combined into an index (with a mean of 0 and SD of 1) using principal components analysis. Higher value represents higher socioeconomic status.

2 Estimated prepregnancy BMI was calculated from estimated prepregnancy weight (based on polynomial regression with gestational age, gestational age squared, and gestational age cubed as predictors) [55] and height at enrollment.

3 Born during the dry season.

### Effect of SQ-LNS on pubertal status

At 9–11 y, SQ-LNS (mean ± SD) did not impact pubertal status in the minimally adjusted (0.01 ± 0.95 compared with non-LNS: −0.01 ± 0.98, *P* = 0.748) or fully adjusted [mean difference (SE): 0.00 (0.06), *P* = 0.958] models. At 11–13 y, the SQ-LNS group was marginally more advanced in the minimally adjusted model (mean ± SD = 0.07 ± 1.04 compared with non-LNS: –0.04 ± 0.98, *P* = 0.078), and the group difference was significant in the fully adjusted model [mean difference (SE): 0.13 (0.07), *P* = 0.049].

### SQ-LNS effect modifiers

**FIGURE 2:**
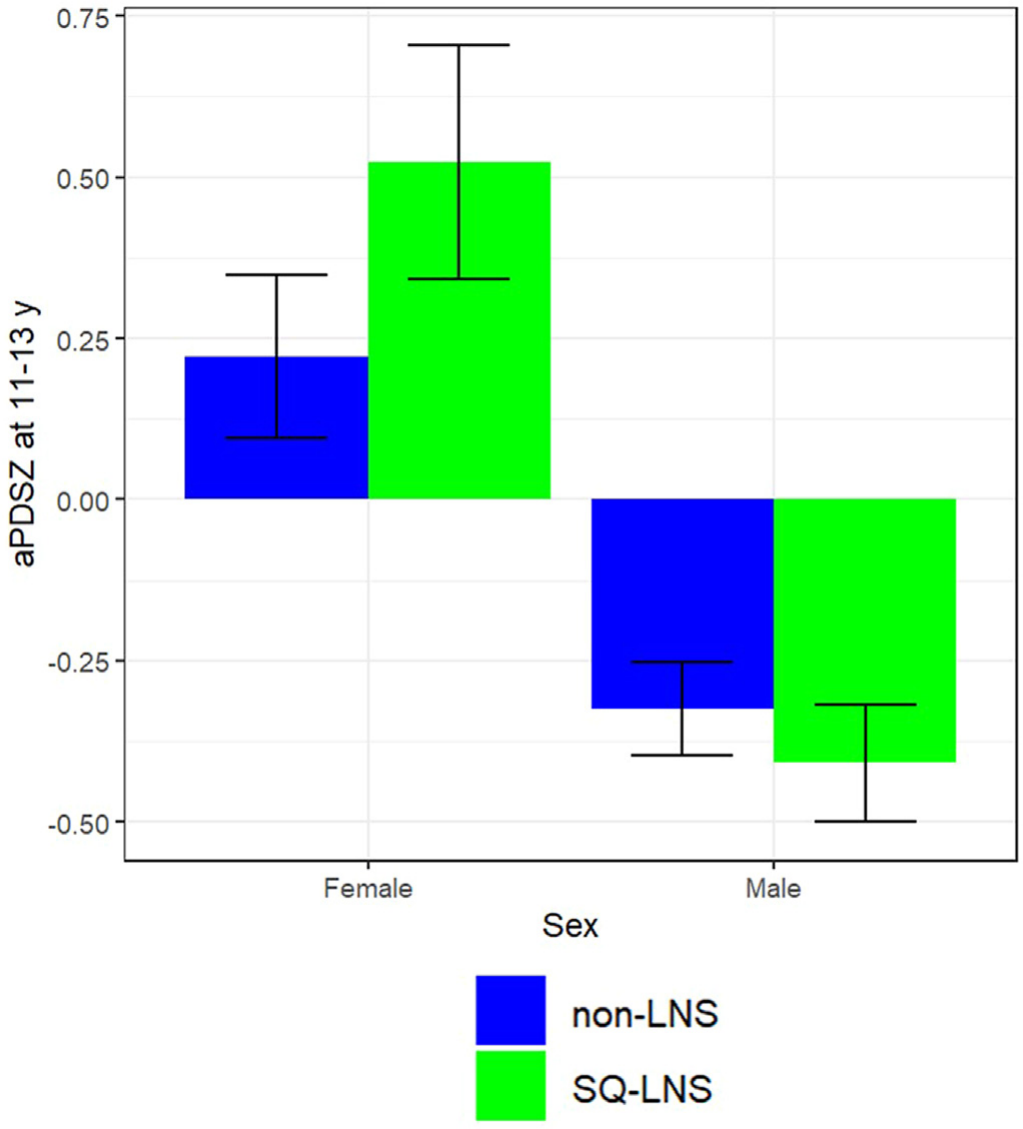
SQ-LNS effect modification by sex at 11–13-y follow-up. aPDSZ, age-adjusted pubertal development scale z-score; non-LNS, iron and folic acid + multiple micronutrient groups; SQ-LNS, small-quantity lipid-based nutrient supplement.

**FIGURE 3:**
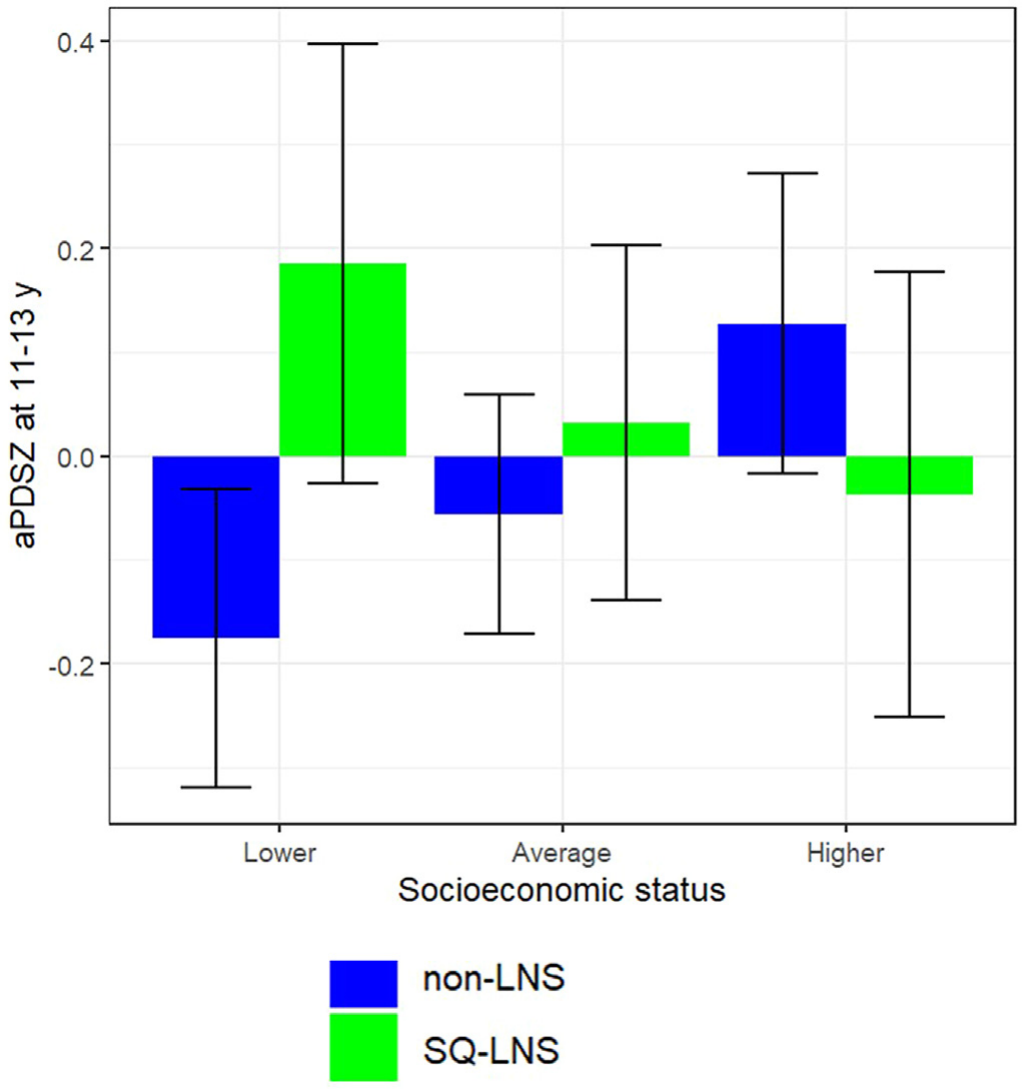
SQ-LNS effect modification by asset index z-score by tertile groups at 11–13-y follow-up. aPDSZ, age-adjusted pubertal development scale z-score; non-LNS, iron and folic acid + multiple micronutrient groups; SQ-LNS, small-quantity lipid-based nutrient supplement.

At 9–11 y of age, sex and household asset index z-score did not modify the effect of SQ-LNS on pubertal development (*P*-interaction > 0.10). At 11–13 y of age, the effect of SQ-LNS on pubertal stage differed by sex (*P*-interaction = 0.003, Figure 2) and household asset index z-score (*P*-interaction = 0.002; Figure 3). More advanced pubertal stage was observed in the SQ-LNS compared with non-LNS group among females (mean difference = 0.30 SD; 95% CI: 0.08, 0.52; *P* = 0.007) but not among males (*P* = 0.167). More advanced pubertal stage was found for SQ-LNS compared with non-LNS groups in the first asset tertile (mean difference = 0.36 SD; 95% CI: 0.13, 0.60; *P* = 0.002) but not in the second tertile (*P* = 0.436) or highest asset tertile (*P* = 0.332) subgroups of household asset index z-score. Birth order did not modify the impact of SQ-LNS on pubertal stage at either age (*P*-interaction > 0.10).

### SQ-LNS effect mediators at 11–13 y

Mediation analysis showed that SQ-LNS impacted aPDSZ partially through birth weight for gestational age z-score (total effect = 0.12 SD, *P* = 0.074; direct effect = 0.11 SD, *P* = 0.098; indirect effect = 0.01 SD, *P* = 0.096; proportion mediated = 7%, *P* = 0.150). Child BMIZ at 4–6, 9–11, and 11–13 y were not associated with SQ-LNS intervention group (*P* > 0.10) and thus were not found to be mediators.

## Discussion

To our knowledge, this is the first RCT to investigate the impact of early life SQ-LNS during the first 1000 d of life on pubertal development. We found that adolescents who received SQ-LNS from 6 to 18 mo of age after maternal SQ-LNS consumption during pregnancy and 6 mo postpartum had greater mean aPDSZ at 11–13 y than those in the non-LNS group who received no direct supplementation after maternal IFA or MMN during pregnancy, and placebo or MMN during 6 mo postpartum.

We did not observe an intervention effect on pubertal stage at 9–11 y. A lack of SQ-LNS effect on pubertal status at 9–11 y of age may be explained by relatively low reproductive maturity at 9–11 y when puberty has barely started, compared with increased reproductive maturity at 11–13 y when puberty is usually underway.

Pubertal development is influenced by an interplay of hormones, central neurotransmitters, and environmental factors [2,56,57] including nutrition, that induce the maturation of the HPG axis, although the mechanism of HPG axis reactivation is unknown [3]. Thus, the activity of the endocrine axes may be altered during the first 1000 d of life by inadequate nutrient supplies to the fetus, decreased nutrient bioavailability, and restricted growth, possibly dysregulating the HPG axis and consequently impacting pubertal status. Because SQ-LNS is a nutrient-dense supplement, it may have contributed to the timely and appropriate regulation of the HPG axis during pregnancy and infancy, preparing the child for the reactivation of the HPG axis during puberty when the neural circuitry established during early life and infancy by gonadal steroids becomes functional again.

Increase in weight is known to be associated with increases in leptin, insulin, IGF-1, and cortisol levels [58,59]. Elevated insulin and IGF-1 levels promote growth in lean body mass, muscle mass, and fat mass, triggering a growth spurt [58]. Leptin and IGF-1 also contribute to skeletal development by stimulating bone mineral accretion and bone maturation [58]. Increased leptin also stimulates hormonal activity in the hypothalamus, pituitary, adrenals, and gonads, expediting pubertal development [58]. However, it is unlikely that the observed group difference in mean aPDSZ at age 11–13 y was a result of group differences in obesity, as we found no intervention effect on BMI at 4–6, 9–11, or 11–13 y. Hence, BMI was not a mediator for pubertal development. Although BMI may not be an adequate proxy for fat mass, in this cohort BMI at 11–13 y was strongly correlated with triceps skinfold (rho = 0.80) and mid-upper arm circumference (rho = 0.94), which suggests that there were no intervention group differences in fat mass at 11–13 y, as was the case at 4–6 y [49] and 9–11 y [45]. Regarding effect modification, we found greater effects of SQ-LNS on aPDSZ among females and among adolescents in households with lower asset index z-scores. This agrees with previous findings indicating that the effect of SQ-LNS on social-emotional difficulties at 4–6 y was greater among children from disadvantaged homes [46], and the effect on height at 9–11 y was greater among females [45]. During infant and child development, males and females show differences in their responses to hormonal changes [60]. Furthermore, reactivation of the HPG axis during adrenarche and gonadarche begins earlier in girls than in boys [60]. Therefore, females may have had greater potential to respond to early life SQ-LNS with regard to pubertal development at this age, compared with males. Because males typically exhibit later pubertal development than females [61], we may see an effect of SQ-LNS on this outcome among males at older ages, that is, >13 y. Regarding significant effect modification in lower socioeconomic households, it is possible that children with lower asset index scores with higher risk of malnutrition [62] and poor cognitive functions [63] tended to respond better to SQ-LNS compared with children from average and higher socioeconomic households. This hypothesis is supported by a prior individual participant meta-analysis [64] wherein greater effects of SQ-LNS was found on language, motor, and executive function among children in lower socioeconomic households. We did not find birth order to be a significant modifier of SQ-LNS impact on pubertal status in this study, even though effects of SQ-LNS on stunting and underweight were greater in later-born than first-born children in individual participant data meta-analyses [33].

Higher birth weight for gestational age z-score partially mediated the effect of SQ-LNS on aPDSZ. Previous studies have investigated birth and early life influences on puberty using anthropometric indices like birth weight as exposures [1,65,66]. Most of these studies have focused on the Tanner stages [65] or a few of the milestones on the PDS [67], and not the total PDS score. For example, in the Dortmund Nutritional and Anthropometric Longitudinally Designed (DONALD) Study [68], it was reported that children who weighed between 2500 and <3000 g at birth were ∼7 mo younger at age at take-off of the pubertal growth spurt than children who weighed ≥3000 g. This appears to conflict with our findings showing that SQ-LNS impacts aPDSZ partially through higher birth weight for gestational age. However, it is difficult to compare our findings with those of the DONALD study as we assessed how birth weight for gestational age, not birth weight, is associated with pubertal development. In this trial, we found that maternal SQ-LNS increased birth weight compared with the IFA group and reduced the risk of SGA compared with the MMN group [44]. Therefore, the mediation by birth weight for gestational age observed herein is plausible, although it explained only 7% of the total effect of SQ-LNS on aPDSZ.

### Study strengths and limitations

Our study has several strengths to note. First, this was an RCT, and the SQ-LNS and the non-LNS groups at 9–13 y did not differ significantly in baseline characteristics except for household asset index z-score, suggesting that the intervention groups remained balanced over time. Second, we had large sample sizes with good retention rates at both the 9–11 y (79%) and 11–13 y (75%) follow-up. Third, we used the PDS (for assessing pubertal stage) that has shown high validity in previous research [50]. There are some potential limitations, however. Because we did not assess pubertal development at 7 y, we may have missed cases of precocious puberty in our study cohort. Consequently, we are unable to determine whether the SQ-LNS induced earlier onset of pubertal development than the non-LNS group. Nevertheless, the results show no record of precocious puberty assessed by menarche among females by self-report, and the mean aPDSZ does not suggest an earlier onset of pubertal development. Another limitation is that participants included in these analyses differed significantly in maternal parity (*P* < 0.05) compared with those lost to follow-up. However, it is unlikely that this led to bias in the results because parity was not associated with pubertal status.

### Conclusions and recommendations

Provision of SQ-LNS in this setting during the first 1000 d of life was associated with more advanced pubertal stage among females at 11–13 y, but not at 9–11 y. Because we did not observe more advanced puberty in the younger age group, the results suggest that the advancement of puberty at 11–13 y was not an adverse outcome. This finding provides additional evidence that SQ-LNS influences both physical and behavioral development. More research is needed to understand the potential impact of early life SQ-LNS exposure on pubertal development among males, and the consequences of more advanced pubertal development among females.

## Author contributions

The authors’ responsibilities were as follows – HN, SAA, ELP, BMO, AM, PDH, AEG, KGD, BA: conceptualized and designed the follow-up study; HN, SAA, ELP, EA, HJB, MOM, XT: conducted the research; HN, CA: conducted the statistical analyses; HN: led the writing of the manuscript with critical review from AM, KGD, CA, SAA, BMO, PDH, AEG, HJB, LMDA, MOM, and FAL. HN: had primary responsibility for final content. Because of her death during the preparation of this manuscript co-author ELP was unable to read and approve the final manuscript; all other authors: read and approved the final manuscript.

## Conflict of interest

The authors report no conflict of interest.

## Funding

This publication is based on research funded by grants to the University of California, Davis, from the National Institutes of Health (R01HD099811) and the Bill & Melinda Gates Foundation (OPP49817). The funders did not play a role in the design or implementation of the study or interpretation of the data. The findings and conclusions contained within are those of the authors and do not necessarily reflect the positions or policies of the Bill & Melinda Gates Foundation.

## Data availability

Data (on de-identified individuals) described in the manuscript, code book, and analytic code will be made available upon request to researchers who provide a methodologically sound proposal and statistical analysis plan contingent on approval by the Principal Investigators. Proposals should be submitted to the corresponding author.
