# Supplementary material for: Impact of Small-Quantity Lipid-Based Nutrient Supplements on Pubertal Status of 9–13-Year Olds: A Follow-Up Study of the iLiNS-DYAD-Ghana Trial
Source: Curr Dev Nutr. 2024 Sep 26;8(12):104458. doi: 10.1016/j.cdnut.2024.104458 (PMC11697765; doi:10.1016/j.cdnut.2024.104458)
Supplement: Multimedia component 1 [file mmc1.docx]

**Supplementary material**

Manuscript Title

Impact of small-quantity lipid-based nutrient supplements on pubertal status of 9–13-year-olds: A follow-up study of the iLiNS-DYAD Ghana trial

First author

Helena Nti

**Supplementary Table 1***.* Comparison of characteristics between those included in the 9-11 y follow-up study versus those lost to follow-up

| **Variable** | | **Included**  n=966  Mean (SD) or  % [n/total] | | **Excluded**  n=354  Mean (SD) or  % [n/total] | | P-value | |
| --- | --- | --- | --- | --- | --- | --- | --- |
| Maternal age, y | | 26.9 (5.4) | | 26.2 (5.7) | | 0.068 | |
| Maternal education, y | | 7.7 (3.6) | | 7.5 (3.9) | | 0.534 | |
| Married or cohabiting, % (n/N) | | 93.3 (901/966) | | 91.2 (323/354) | | 0.255 | |
| Household asset index z-score^1^ | | 0.03 (0.97) | | -0.08 (1.06) | | 0.095 | |
| Household food secure, % (n/N) | | 57.9 (559) | | 54.5 (193/336) | | 0.939 | |
| Household improved water source, % (n/N) | | 98.3 (950/965) | | 91.8 (325/340) | | 1.000 | |
| Household toilet facility, % (n/N) | | 97.4 (941/964) | | 90.7 (321/331) | | 0.667 | |
| Maternal height, cm | | 158.9 (5.7) | | 158.6 (5.6) | | 0.521 | |
| Pre-pregnancy BMI^2^, kg/m^2^ | | 24.5 (4.4) | | 24.5 (4.2) | | 0.979 | |
| Hemoglobin concentration, g/L | | 111.4 (12.1) | | 111.3 (11.9) | | 0.844 | |
| Primiparous, % (n/N) | | 31.5 (304/966) | | 40.1 (142/354) | | 0.004 | |
| Child female | | 51.7 (499/966) | | 38.7 (137/283) | | 0.372 | |

Marital status was different between the groups at 11-13 y (*P* = 0.031).

^1^Proxy indicator for household socioeconomic status constructed for each household based on ownership of a set of assets (radio, television etc.), lighting source, drinking water supply, sanitation facilities, and flooring materials. Household ownership of this set of assets is combined into an index (with a mean of zero and standard deviation of one) using principal components analysis. Higher value represents higher socioeconomic status. ^2^Estimated pre-pregnancy BMI was calculated from estimated pre-pregnancy weight (based on polynomial regression with gestational age, gestational age squared, and gestational age cubed as predictors) (73) and height at enrollment.
